# Intradialytic Hypotensive Episodes are Only Occasionally Associated With Adverse Symptoms

**DOI:** 10.1016/j.ekir.2025.103718

**Published:** 2025-12-12

**Authors:** Sabrine Chaara, Paul A. Rootjes, Miquéla I.Y. Bergtop, Muriel P.C. Grooteman, Peiyun Liu, Menso J. Nubé, Gertrude Wijngaarden, Camiel L.M. de Roij van Zuijdewijn

**Affiliations:** 1 Division of Nephrology, Department of Internal Medicine, Amsterdam UMC, Amsterdam, The Netherlands; 2 Amsterdam Cardiovascular Sciences, Diabetes and Hypertensive Diseases, Amsterdam, The Netherlands; 3 Department of Internal Medicine, Gelre Hospitals, Apeldoorn, The Netherlands; 4 Department of Renal Medicine, Singapore General Hospital, Singapore; 5 Department of Internal Medicine, Spaarne Gasthuis, Haarlem, The Netherlands

**Keywords:** blood pressure, hemodiafiltration, hemodialysis, intradialytic hypotension, patient-reported outcome measures, symptoms

## Abstract

**Introduction:**

Although intradialytic hypotension (IDH) is a frequent complication of hemodialysis (HD), a consensus definition is lacking. Adverse symptoms and/or interventions are a prerequisite in guidelines; however, a threshold-based blood pressure (BP) definition appeared most strongly associated with mortality. In this study, we evaluated the association of IDH with both real-time symptoms and physical intradialytic patient-reported outcome measures (PID-PROMs) in prevalent HD and hemodiafiltration (HDF) patients.

**Methods:**

This is a secondary analysis of the HOLLANT study, a randomized cross-over trial comparing 4 dialysis modalities (i.e., standard HD, cool high-flux HD, low-volume HDF, and high-volume HDF), each applied for 2 weeks in 40 patients. In each second treatment week, BP was measured every 15 minutes and when symptoms occurred. Symptoms were documented real-time and PID-PROMs were collected after each modality. Considering that symptoms occurred independent of dialysis mode, data from all treatment modalities were pooled. IDH was threshold-defined (systolic BP [SBP] < 90 or < 100 mm Hg, dependent on predialysis SBP). The incidence of symptomatic IDH (sIDH) and asymptomatic IDH (aIDH) was assessed, with sIDH defined by concurrent symptoms. When real-time symptoms arose, attribution to IDH was appraised according to the threshold, National Kidney Disease Outcomes Quality Initiative, and the European Best Practice Guideline definitions. Differences in PID-PROMs were analyzed in tertiles of IDH occurrence.

**Results:**

In 20.1% of 458 treatments, 222 IDH episodes were observed (0.48/session). The majority occurred toward the end of dialysis and 98% was asymptomatic. Real-time reported symptoms (*n* = 24) were noted in 5.2% of the sessions. Although half were BP-related (*n* = 13/24), just 4 fulfilled the threshold criteria. Associations between IDH and PID-PROMs were largely absent.

**Conclusion:**

aIDH exceeds sIDH by far. Therefore, symptom-based definitions severely underestimate aIDH incidence. Both real-time occurring symptoms and PID-PROMs are only occasionally associated with IDH.

## Graphical abstract

**Figure:**
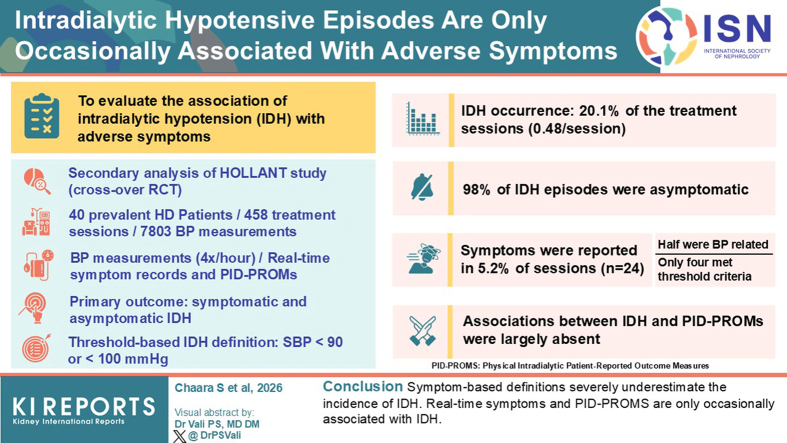

IDH is a frequent complication of intermittent extracorporeal renal replacement therapy.^1^, ^2^, ^3^, ^4^ Depending on its definition, the prevalence varies between 4% and 50%.^2^^,^^5^ Intradialytic BP decreases may occur when the ultrafiltration (UF) rate surpasses the plasma refill rate and compensatory mechanisms, such as an increase in arteriolar tone and cardiac output, are insufficient.^3^^,^^6^ As a result, BP declines, which may cause tissue ischemia,^3^^,^^6^ adverse symptoms,^4^^,^^7^ and even mortality.^2^^,^^8^ To alleviate these complications, in daily practice, UF rate is reduced, fluid is administered, and/or treatment is interrupted. As a result, undertreatment may occur, resulting influid overload, inadequate removal of uremic toxins, and/or insufficient correction of acid-base disorders.^9^

Despite its high incidence, a universally accepted definition of IDH is lacking. Not only the magnitude of BP declines, but also the occurrence of symptoms and/or the need for interventions differs between definitions.^4^^,^^10^^,^^11^ Given that data on symptoms and interventions are usually not stored in large databases, several IDH definitions rely solely on BP measurements.^3^ However, in clinical practice, BP is only measured twice/hour, whereas additional readings and/or interventions are carried out when symptoms arise. Therefore, it may not be surprising that the presence of symptoms became a prerequisite for the IDH definitions of the National Kidney Disease Outcomes Quality Initiative (KDOQI) and the European Best Practice Guideline (EBPG).^12^^,^^13^ A comprehensive retrospective study analyzing the associations between 8 commonly used IDH definitions and mortality in nearly 12,000 patients, found that an absolute SBP < 90 mm Hg or < 100 mm Hg (with a predialysis SBP < 160 mm Hg or SBP ≥ 160 mm Hg, respectively) is most strongly associated with mortality. According to this investigation, the adjusted odds ratios for 1-year and 2-year mortality were 1.30 (95% confidence interval: 1.07–1.57) and 1.56 (95% confidence interval: 1.05–2.31), respectively. IDH definitions requiring symptoms and/or interventions did not correlate with clinical outcome at all.^2^

Given the absence of well-executed studies and the wide variation in data collection, it is unclear whether, and to which extent, IDH and adverse symptoms are interrelated. In case of a strong relation, asymptomatic IDH (aIDH) occurs sporadically. When the relation is weak and only symptomatic IDH (sIDH) episodes are noted, many aIDH episodes will be missed. As mentioned earlier, overlooking IDH may have a negative impact on the outcome of our patients.

Given that repetitive IDH, with or without adverse symptoms, may contribute to ischemic complications, and current guidelines require symptoms for its diagnosis, we conducted a secondary analysis of the HOLLANT study^14^ (NCT03249532) to assess the relationship between IDH and symptoms in adult patients on chronic dialysis in 3 ways. First, we evaluated the incidence of sIDH and aIDH as defined by the threshold-based criteria. Second, we quantified the proportion of real-time reported symptoms related to IDH, according to the following 3 definitions: threshold-based, and the KDOQI and EBPG guidelines. Lastly, we compared the frequency and severity of PID-PROMs, between groups of IDH-prone, IDH-intermediate and IDH-resistant individuals.

## Methods

### Study Design

In this analysis, the relationship between IDH and adverse symptoms was assessed as a prespecified secondary objective of the HOLLANT study (ClinicalTrials.gov identifier NCT03249532), which was an open-label, multicenter randomized cross-over trial in chronic dialysis patients. For the present analyses, data from 4 treatment modalities were pooled, because we demonstrated previously that PID-PROMs are unaffected by dialysis modality.^15^ The design of this study has been described extensively elsewhere.^14^ In short, patients were exposed to the following: (i) standard high-flux HD (dialysate temperature [T_d_]: 36.5 °C), (ii) cool high-flux HD (C-HD) (T_d_: 35.5 °C), (iii) low-volume postdilution HDF (target convection volume: 15 L/session, T_d_: 36.5 °C), and (iv) high-volume postdilution HDF (target convection volume ≥ 23 L/session, T_d_: 36.5 °C), each for 2 weeks (Supplementary Figure S1). Because the first week of each modality served as a wash-out period, all measurements were conducted during the second week. All patients provided written consent. The study was conducted in accordance with the Declaration of Helsinki and approved by the Medical Ethical committee of VU University Medical Center (METC VUmc: 2017.581/ NL61210.029.17).

### Study Population

Patients were recruited from an independent clinic (Niercentrum aan de Amstel, Amstelveen, The Netherlands), a large community hospital (St. Antonius hospital, Nieuwegein, The Netherlands) and an academic hospital (Amsterdam UMC, location VU University medical center, Amsterdam, The Netherlands) from July 2018 to February 2021. Patients with a dialysis vintage > 2 months were considered eligible, if they were treated with HD or HDF 3 times/wk for ≥ 4 hours per session. Other inclusion criteria were as follows: ability to understand the study, willingness to provide informed consent, single-pool Kt/V_urea_ ≥ 1.2, access recirculation < 10%, and a blood flow rate ≥ 350 ml/min and/or ability to achieve a CV ≥ 23 L per treatment. Exclusion criteria were as follows: age < 18 years, life expectancy < 3 months, participation in another intervention trial or severe noncompliance to the dialysis procedure and accompanying prescriptions.

### Dialysis Prescription and Equipment

All sessions lasted 4 hours and were performed with Xevonta 23 high-flux dialyzers on Dialog iQ dialysis machines, mantled with captive lines DiaStream iQ (all B. Braun Avitum AG, Melsungen, Germany). HDF was conducted online in the postdilution mode. Blood flow rate was targeted at 350 to 400 ml/min, and filtration fraction (blood flow rate/convection flow rate) was maintained at 25% to 30% for high-volume postdilution HDF. Ultrapure dialysis fluids (< 0.1 colony forming units/ml, < 0.03 endotoxin units/ml) were mixed using Sol-Cart Bicarbonate cartridges and acidic dialysate (all B. Braun Avitum AG, Melsungen, Germany). Substitution fluid was prepared from the dialysis fluid via an additional UF-step, using a fluid filter (Diacap Ultra, B. Braun Avitum AG, Melsungen, Germany), before infusing into the bloodstream. Treatment settings were kept consistent for each patient throughout all modalities. Patients received their usual dosage of low molecular weight heparin, which was adjusted when needed. Routine patient care was provided in accordance with current guidelines.^12^^,^^16^

### IDH Assessment (Threshold-Based Definition)

IDH was defined as SBP < 90 mm Hg for a predialysis SBP < 160 mm Hg or SBP < 100 mm Hg for a predialysis SBP ≥ 160 mm Hg, according to threshold-based criteria.^2^ In the second week of each modality, BP was measured with an automated manometric cuff device connected to the machine (automatic BP monitor, B. Braun Avitum AG, Melsungen), both at the start and every 15 minutes thereafter, during 3 sessions. Each IDH episode was classified as sIDH or aIDH, depending on the presence of concurrent symptoms (Supplementary Figure S2).

### Registration and Assessment of Adverse Symptoms

After providing clear instructions to the patients, adverse symptoms were evaluated in 2 ways as follows: (i) real-time, during 3 sessions in the second week, coinciding or not with the 15-minute BP measurements; (ii) PID-PROMs immediately after the last session in the second week, using the modified Dialysis Symptom Index (DSI, mDSI) questionnaire (Supplementary Table S1).^15^

#### Real-Time Reported Adverse Symptoms

When real-time adverse symptoms occurred outside the regular 15-minute BP readings, extra BP measurements were performed. All real-time reported symptoms were evaluated for concurrent IDH using the following 3 definitions (Supplementary Figure S2): (i) the threshold-based definition,^2^ (ii) the KDOQI definition (SBP decrease ≥ 20 mm Hg or mean arterial pressure decrease ≥ 10 mm Hg accompanied by symptoms, i.e., abdominal discomfort, yawning, sighing, nausea, vomiting, muscle cramps, restlessness, dizziness, fainting, or anxiety),^12^ and (iii) the EBPG definition (KDOQI description plus intervention).^13^ If a real-time reported symptom was accompanied by IDH according to the threshold-based definition, provided that the symptom was indicative of BP decrease (as listed in the KDOQI symptom criteria), it was denoted as sIDH. If any definition was met, the symptom was categorized as BP-related.

#### PID-PROMs

PID-PROMs experienced in the second week (i.e., 3 sessions) were evaluated with the mDSI (Supplementary Table S1),^15^ which is the DSI restricted to physical symptoms and extended with the items “feeling cold”, “shivering”, and “recovery time”.^7^^,^^17^^,^^18^ One week before entering the study, patients were trained in how to complete the questionnaire. In addition to oral and visual explanation of the mDSI, the teach-back method of the survey items was applied,^19^ which was reiterated at the first assessment. The following parameters were derived from the mDSI, which was completed immediately after the last session of each modality (i.e., 4 questionnaires/patient):

##### Frequency and Severity of Discrete Symptoms and Recovery Time

The mDSI employed consists of 13 items; 12 discrete symptoms, supplemented with the topic recovery time. Patients reported whether they experienced the symptom in the past week (0 = absent) and, if present, rated its severity by using a Likert scale ranging from 1 (a little bit) to 4 (very much). Recovery time ranged from 0 (direct recovery) to 4 (until the next session).

##### Total Number of Symptoms

The total number of symptoms was calculated by summing all side effects, each consisting of 0 to 12 items (excluding “recovery time”). The mean number of symptoms per questionnaire was averaged for each subject.

##### Overall Symptom Severity Score

The overall symptom severity score per patient was calculated by summing up the severity scores (1–4) and dividing them by the total number of reported symptoms. The median overall severity scores of the 4 questionnaires were calculated for each subject.

##### Symptom Burden

Symptom burden was calculated by aggregating the severity scores (1–4) of all reported symptoms (0–12), yielding a range of 1 to 48. For each individual patient, the symptom burden-score was averaged.

### IDH Susceptibility

The IDH incidence per patient was calculated by dividing the cumulative number of IDH episodes (threshold defined) by the total number of BP readings. Based on these scores, patients were stratified into tertiles of IDH susceptibility (IDH resistant, IDH intermediate prone, and IDH prone).

### Statistical Methods

Descriptive statistics were summarized as mean ± SD for normally distributed continuous variables, median with interquartile range for nonnormally distributed continuous variables, and counts with percentages for categorical variables.

Data from all treatment modalities were pooled to examine the relationship between IDH and adverse symptoms, because we previously demonstrated that PID-PROMs are unaffected by dialysis modality (with the exception of cold feelings).^15^ Stratified data of IDH in relation to both real-time symptoms and PID-PROMs by dialysis modality are provided in the Supplementary Material and presented descriptively because of the small subgroup sizes.

An area-proportional Venn diagram was used to illustrate the relationship between IDH and symptomatic events.

To analyze symptoms among tertiles, their occurrence was dichotomized for each patient (i.e., present in ≥ 1 of the questionnaires or absent) and logistic regression models using IDH-resistant patients as reference category were used. Kruskal-Wallis tests were used to compare the median severity score for each discrete symptom, the total number of symptoms, symptom burden, and recovery time across IDH tertiles. The relation between the IDH frequency and symptom burden was analyzed using a scatter plot and Kendall’s Tau Correlation test. A 2-sided *P*-value < 0.05 was considered significant. Analyses were performed with SPSS (version 28, IBM Inc., NY).

## Results

### Patient and Treatment Characteristics

As shown in Supplementary Figure S3, 45 patients were enrolled, but 5 dropped out before randomization. Baseline characteristics are summarized in Table 1. Forty patients completed the study, of whom 75% were male, with a mean age of 69.7 ± 13.5 years. Median dialysis vintage was 3.0 (interquartile range: 1.0–5.8) years. Diabetes mellitus was present in 48% and cardiovascular disease in 73% of the patients. Dialysis characteristics are shown in Supplementary Table S2. Overall, mean UF volume was 2.3 ± 0.7 L per session and UF rate was 7.7 ± 2.1 ml/kg/h. The median intraindividual target-weight variation was 0.07 (interquartile range: 0.0–0.2) kg.

**Table 1:** Patient characteristics

Characteristics	All patients (*N* = 40)
Demographics	
Sex (male)	30 (75%)
Age (yrs)	69.7 (13.5)
Ethnicity	
Caucasian/African/Asian	28 (70%)/ 10 (25%)/ 2 (5%)
Clinical characteristics	
BMI (kg/m^2^)	26.7 (4.2)
Smoking status	
Nonsmoker/ former smoker/ current smoker	14 (35%)/ 18 (45%)/ 8 (20%)
Systolic blood pressure, predialysis (mm Hg)	145 (23)
Diastolic blood pressure, predialysis (mm Hg)	81 (13)
Residual kidney function^a^	24 (60%)
Residual kidney function (ml/min)^b^	1.9 (1.0–2.5)
Medical history	
Dialysis modality	
HD/HDF	17 (42.5%)/ 23 (57.5%)
Dialysis vintage (yrs)	3.0 (1.0–5.8)
History of kidney transplantation	3 (7.5%)
Primary cause of ESKD	
Glomerulonephritis	10 (25%)
Renal vascular disease	9 (22.5%)
Diabetic nephropathy	15 (37.5%)
Cystic kidney disease	1 (2.5%)
Other/Unknown	4 (10%) / 1 (2.5%)
Comorbid conditions	
Diabetes mellitus	19 (47.5%)
Hypertension	28 (70%)
History of CVD	29 (72.5%)
Heart failure	18 (45%)
Myocardial infarction	11 (27.5%)
Angina pectoris	11 (27.5%)
CVA/TIA	9 (22.5%)
Peripheral artery disease	8 (20%)
Medication	
ACE-I/ARB	10 (25%)
Beta blocker	25 (62.5%)
Calcium antagonist	10 (25%)
Diuretic	11 (27.5%)

ACE-I, angiotensin-converting enzyme inhibitor; ARB, angiotensin II receptor blocker; BMI, body mass index; CVA, cerebral vascular accident; CVD, cardiovascular disease; ESKD, end-stage kidney disease; HD, hemodialysis; HDF, hemodiafiltration; TIA, transient ischemic attack.

Values are presented as number (*n*) (%) for categorical variables and mean (SD) or median (interquartile range) for continuous variables.

a Residual diuresis > 100 ml/24 h.

b In patients with diuresis > 100 ml/24 h.

### Missing Data

Four patients did not complete all treatments: 2 were not exposed to HDF because of technical issues and 2 withdrew consent after finishing 50% and 75% of the study, respectively. Although BP measurements were obtained in 458 treatments, 5.1% out of 7803 BP readings were missing. One hundred fifty questionnaires (94%) were completed. Missing surveys were due to nonparticipation in a certain treatment modality.

### IDH (Threshold-Based Definition)

**Figure 1:**
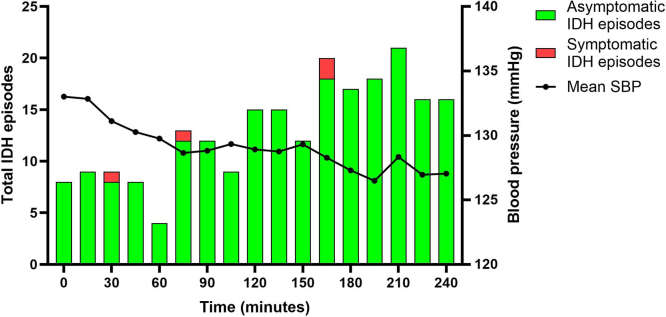
Frequency of symptomatic and asymptomatic IDH. Overview of symptomatic and asymptomatic IDH episodes during 458 dialysis treatments. IDH was defined as SBP < 90 mm Hg or < 100 mm Hg with a predialysis SBP < 160 mm Hg or SBP ≥ 160 mmHg, respectively. IDH, intradialytic hypotension; SBP, systolic blood pressure (mm Hg).

Mean predialysis SBP and DBP decreased from 133 ± 15 and 78 ± 12 mm Hg to 127 ± 15 and 75 ± 11 mm Hg postdialysis, respectively. According to the threshold definition, 222 IDH episodes occurred in 92 out of 458 sessions (20.1%), with an average of 0.48 IDH episodes per treatment. IDH frequency within each treatment modality has been reported previously.^20^ In Supplementary Table S3, we provide the distribution of aIDH and sIDH episodes across modalities. In 75% of patients, ≥ 1 IDH episodes were observed. The IDH frequency increased toward the end of sessions, and 98.2% were aIDH (218/222; Figure 1). Real-time reported symptoms were observed in only 4 IDH episodes (sIDH: 4/222 [1.8%]), manifested as dizziness (*n* = 3/4), sweating (*n* = 2/4), loss of consciousness (*n* = 1/4), nausea (*n* = 1/4), and yawning (*n* = 1/4). Three out of 4 sIDH episodes occurred during standard high-flux HD, whereas 1 was observed in high-volume postdilution HDF (Supplementary Table S3).

### Adverse Symptoms

#### Real-Time Intradialytic Symptoms

**Figure 2:**
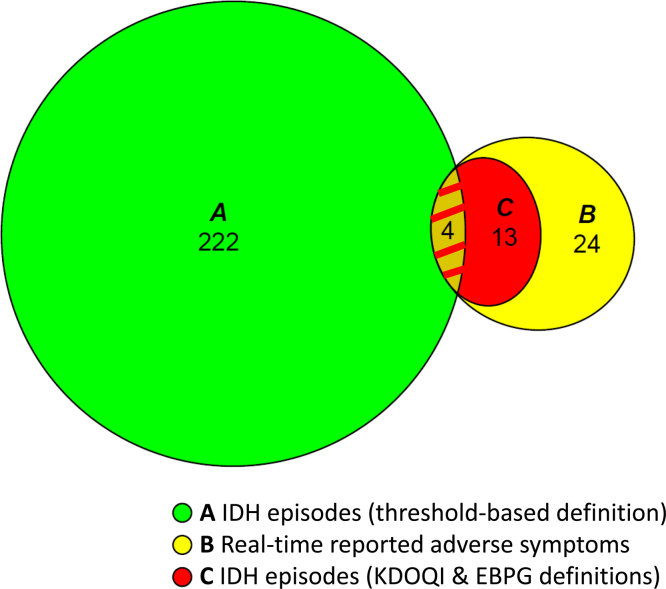
Association between IDH and adverse symptoms. Proportional Venn-diagram: (a) 222, (b) 24, (c) 13 out of 24 real-time symptoms fulfilled the KDOQI and EBPG IDH definitions. Four out of 24 real-time reported adverse symptoms fulfilled all IDH definitions (a, b, c). Threshold-based definition: SBP < 90 mm Hg or < 100 mm Hg with a predialysis SBP < 160 mm Hg or SBP ≥ 160 mm Hg, respectively. KDOQI guideline definition: SBP decrease ≥ 20 mm Hg or mean arterial pressure decrease ≥ 10 mm Hg accompanied by symptoms. EBPG definition: KDOQI description + intervention. EBPG, European Best Practice Guideline; KDOQI, National Kidney Disease Outcomes Quality Initiative; IDH, intradialytic hypotension.

Symptoms occurred in 24 out of 458 sessions (5.2%) in 12 out of 40 patients. Only 3 extra BP readings were performed, because most cases coincided with the 15-minute BP monitoring regimen. As mentioned earlier under the heading "Intradialytic Hypotension,“ only 4 (*n* = 4/24) fulfilled the threshold-based IDH criteria. When applying the KDOQI and EBPG IDH definitions, approximately half (13/24) met these criteria, leaving 11 cases unrelated to BP (Figure 2). Dizziness (*n* = 8), muscle cramps (*n* = 8), sweating (*n* = 6), malaise (*n* = 2), and nausea (*n* = 2) were most frequently reported. As shown in Supplementary Table S4, most symptoms were reported during standard high-flux HD (*n* = 10/24) and C-HD (*n* = 6/24), of which 50% was BP-related.

#### PID-PROMs

According to the mDSI, fatigue was reported at least once in 80% of the patients, followed by feeling cold (73%), itching (65%), dizziness (55%), and muscle cramps (30%).

#### IDH Susceptibility and PID-PROMs

Patients were divided into IDH tertiles based on the percentage of BP readings meeting the threshold-based criteria: IDH resistant (< 7.1%), IDH intermediate (7.1%–18.8%), and IDH prone (> 18.8%). Differences in patient characteristics between groups were not observed (Supplementary Table S5).

##### Frequency and Severity of Discrete Symptoms

Compared with IDH-resistant patients, IDH-prone persons reported headaches more often (odds ratio: 12.4 [95% confidence interval: 1.8–83.8], *P* = 0.01). None of the other symptoms were related to IDH susceptibility (Table 2).

**Table 2:** Association between IDH-susceptibility and PID-PROMs

PID-PROMs	IDH resistant (*n* = 13) Reference	Intermediate IDH prone (*n* = 14)			IDH prone (*n* = 13)		
	*n* (%)	*n* (%)	OR (95% CI)	*P*-value	*n* (%)	OR (95% CI)	*P*-value
Dizziness	5 (38%)	9 (64%)	2.88 (0.60–13.75)	0.19	8 (62%)	2.56 (0.53–12.43)	0.24
Nausea	2 (15%)	4 (29%)	2.20 (0.33–14.73)	0.42	2 (15%)	1.0 (0.12–8.42)	1.00
Vomiting	0 (0%)	2 (14%)	n/a		0 (0%)	n/a	
Headache	2 (15%)	5 (36%)	3.06 (0.48–19.66)	0.24	9 (69%)	12.38 (1.83–83.77)	0.01
Muscle cramps	8 (62%)	11 (79%)	2.29 (0.42–12.50)	0.34	9 (69%)	1.41 (0.28–7.13)	0.68
Swelling of the legs	1 (8%)	4 (29%)	4.80 (0.46–50.16)	0.19	5 (38%)	7.50 (0.73–76.77)	0.09
Shortness of breath	3 (23%)	5 (36%)	1.85 (0.34–10.05)	0.48	5 (38%)	2.08 (0.38–11.48)	0.40
Chest pain	5 (38%)	1 (7%)	0.12 (0.01–1.25)	0.08	3 (23%)	0.48 (0.09–2.65)	0.40
Itching	8 (62%)	11 (79%)	2.29 (0.42–12.50)	0.34	7 (54%)	0.73 (0.15–3.47)	0.69
Feeling cold	11 (85%)	11 (79%)	0.67 (0.09–4.80)	0.69	7 (54%)	0.21 (0.03–1.36)	0.10
Shivering	7 (54%)	8 (57%)	1.14 (0.25–5.22)	0.86	3 (23%)	0.26 (0.05–1.39)	0.12
Feeling tired or lack of energy	10 (77%)	12 (86%)	1.80 (0.25–12.99)	0.56	10 (77%)	1.00 (0.16–6.20)	1.00

CI, confidence interval; IDH, intradialytic hypotension; n/a, not applicable; OR, odds ratio; PID-PROMs, physical intradialytic patient-reported outcome measures.

Data are presented as number of patients experiencing the symptom, *n* (%) and OR (95% CI).

Groups are sorted by tertiles of IDH incidence. IDH-resistant patients served as the reference group.

In Table 3, we show the median severity scores of all discrete symptoms, stratified by IDH tertiles. Compared with IDH-resistant and intermediate patients, IDH-prone persons experienced an increased severity of dizziness (1.0 [1.0–1.0] and 1.0 [1.0–2.0] vs. 1.5 [1.0–4.0], respectively; *P* = 0.03).

**Table 3:** IDH susceptibility and symptom severity (PID-PROMs)

PID-PROMs	IDH-resistant (*n* = 13)		Intermediate IDH-prone (*n* = 14)		IDH prone (*n* = 13)		*P*-value
	*n* (%)	Median (range)	*n* (%)	Median (range)	*n* (%)	Median (range)	
Dizziness	5 (38%)	1.0 (1.0–1.0)	9 (64%)	1.0 (1.0–2.0)	8 (62%)	1.5 (1.0–4.0)	0.03
Nausea	2 (15%)	2.0 (2.0–2.0)	4 (29%)	1.0 (1.0–1.0)	2 (15%)	1.0 (1.0–2.0)	0.08
Vomiting	0 (0%)	n/a	2 (14%)	1.0 (1.0–1.0)	0 (0%)	n/a	n/a
Headache	2 (15%)	1.0 (1.0–1.7)	5 (36%)	1.0 (1.0–3.0)	9 (69%)	1.0 (1.0–1.3)	0.68
Muscle cramps	8 (62%)	1.0 (1.0–1.5)	11 (79%)	1.3 (1.0–2.7)	9 (69%)	1.0 (1.0–2.5)	0.17
Swelling of the legs	1 (8%)	1.0 (1.0–1.0)	4 (29%)	1.4 (1.0–2.0)	5 (38%)	1.0 (1.0–1.0)	0.04
Shortness of breath	3 (23%)	1.0 (1.0–2.0)	5 (36%)	1.0 (1.0–2.0)	5 (38%)	1.0 (1.0–2.0)	0.98
Chest pain	5 (38%)	1.0 (1.0–3.0)	1 (7%)	1.0 (1.0–1.0)	3 (23%)	1.0 (1.0–1.0)	0.76
Itching	8 (62%)	1.0 (1.0–2.0)	11 (79%)	1.0 (1.0–2.0)	7 (54%)	1.0 (1.0–2.3)	0.82
Feeling cold	11 (85%)	1.3 (1.0–2.0)	11 (79%)	1.3 (1.0–3.5)	7 (54%)	1.3 (1.0–2.0)	0.68
Shivering	7 (54%)	1.0 (1.0–1.3)	8 (57%)	1.0 (1.0–2.0)	3 (23%)	1.5 (1.0–1.7)	0.19
Feeling tired or lack of energy	10 (77%)	1.7 (1.0–4.0)	12 (86%)	1.4 (1.0–3.7)	10 (77%)	1.1 (1.0–3.0)	0.57
Recovery time	13 (100%)	1.5 (0–3.0)	14 (100%)	1.5 (0–4.0)	13 (100%)	1.0 (0–3.0)	0.33

IDH, intradialytic hypotension; n/a, not applicable; PID-PROMs, physical intradialytic patient-reported outcome measures.

Data are presented as number of patients experiencing the symptom, *n* (%) and median severity score (range: minimum–maximum).

Groups are sorted by IDH incidence.

##### Number of Symptoms, Overall Symptom Severity Score, and Symptom Burden

With respect to the total number of symptoms, overall symptom severity score and symptom burden (Table 4), differences were not observed between IDH-subgroups. In Supplementary Figure S4, we illustrate the lack of association between IDH frequency and mean symptom burden.

**Table 4:** IDH-susceptibility and PID-PROMs, as indicated by the total number of symptoms as well as symptom burden

PID-PROMs	IDH-resistant (*n* = 13)	Intermediate IDH-prone (*n* = 14)	IDH-prone (*n* = 13)	*P*-value
Total number of symptoms	2.4 (1.5–3.6)	2.9 (1.8–4.5)	1.8 (1.3–4.6)	0.82
Overall symptom severity score	1.3 (1.2–1.6)	1.3 (1.1–1.7)	1.3 (1.0–1.7)	0.79
Symptom burden	3.0 (1.9–5.3)	4.4 (2.6–8.2)	3.0 (1.6–5.6)	0.38

IDH, intradialytic hypotension; PID-PROMs, physical intradialytic patient-reported outcome measures.

Data are presented as median (interquartile range). Groups are sorted by IDH incidence.

##### IDH Susceptibility and PID-PROMs Stratified by Dialysis Modality

Additional data stratified by both IDH susceptibility and dialysis modality are provided in Supplementary Tables S6 to S8. No marked differences in PID-PROMs were observed between dialysis modalities across the IDH subgroups.

## Discussion

From this study, it appears that one-fifth of the sessions were complicated by IDH, of which the vast majority (98.2%) passed unnoticed. Furthermore, we found that 75% of the patients experienced ≥ 1 IDH episode in 12 dialysis sessions, with a mean of 0.48 per session. With respect to the course of IDH, a notable increase was observed toward the end of the sessions. Considering the occurrence of adverse symptoms, only 24 real-time reported symptomatic events were noted in 5.2% of the treatments, manifesting in 12 patients. Although over half of these cases were associated with a BP decline as defined by the KDOQI and EBPG guidelines, only 4 met the threshold-based criteria. Moreover, it appeared that PID-PROMs are largely unrelated to IDH susceptibility. Only headache and dizziness were more pronounced in IDH-prone patients. Lastly, the relationship between IDH and adverse symptoms appeared independent of dialysis modality.

A survey on the frequency of IDH-induced symptomatology in previous studies reveals that the number of studies is limited and that the incidence of sIDH varies markedly. It should be recognized, however, that comparison is hindered by methodological differences, including the IDH definitions applied, the timing and frequency of BP readings, and/or the design of the investigations (prospective vs. retrospective). Although a large prospective study showed that 78% of the sessions were complicated by IDH (decrease in SBP ≥ 20 mm Hg or mean arterial pressure ≥ 10 mm Hg), of which 58.2% were asymptomatic,^21^ another investigation found that just a quarter of the asymptomatic patients experienced a SBP < 100 mm Hg.^10^ Quite contrasting, data were reported by others, who retrospectively found that aIDH (decrease in SBP ≥ 20 mm Hg) occurred in only 3.7% of the sessions and sIDH in 9.7%.^22^ Being aware that extra BP readings are generally only performed when symptoms arise, it is in fact, hardly surprising that sIDH outnumbers aIDH in the latter study.

However, by collecting the data prospectively and measuring BP twice as frequent as in most other studies, here we clearly show that aIDH exceeds sIDH by far in daily practice. Its increase over time supports the concept that IDH is mainly caused by volume depletion and/or insufficient compensatory mechanisms to maintain adequate systemic BP. However, because a minority of IDH episodes occurred shortly after the start of extracorporeal renal replacement therapy—when UF is (practically) 0—other mechanisms, such as bio-incompatibility issues,^23^ rapid osmolality or electrolyte shifts, and/or blood sequestration in the extracorporeal circuit seem to be involved as well.^6^^,^^24^

As for the real-time reported symptoms, only 5.2% of the sessions were complicated by side effects, occurring in one-third of the patients. Although about half of these symptoms (*n* = 13/24) were associated with IDH as formulated in the EBPG and KDOQI guidelines, only 4 met the threshold-based criteria. Previously, not only was a higher incidence of adverse symptoms reported (21.4%), but also a much stronger association with IDH (86%).^21^ Our findings suggest that factors beyond BP decreases play a role in the development of dialysis-related symptoms. Although the pathophysiology remains incompletely understood, potential contributing factors include rapid biochemical changes, cardiac comorbidities (such as cardiac stunning and arrythmias), differences between patient and T_d_, and dialysis-related anxiety.^21^^,^^25^ Importantly, hemodynamic stress can occur even in the absence of a significant BP drop.^10^^,^^21^

Considering that the factual relation between IDH and side effects was unknown, the third objective of this study was to establish or refute their supposed association in patient groups with varying IDH susceptibility. Consistent with earlier literature, IDH-prone patients experienced headaches and dizziness more severely than IDH-resistant persons did.^26^ With respect to the remaining discrete symptoms, total symptom count, symptom severity, and symptom burden, however, PID-PROMs did not differ. However, the likelihood of individuals with IDH developing adverse symptoms was greatest. Thus, although the majority of IDH episodes occurred asymptomatic, the latter finding indicates that IDH and adverse symptoms are occasionally related.

Considering that the dialysis modality may affect both intradialytic hemodynamics and symptom occurrence, we explored the relationship between IDH and symptoms across dialysis modalities. As previously reported, IDH occurred least frequently during C-HD and high-volume postdilution HDF,^20^ which is possibly mediated by an increase in total peripheral resistance and interstitial fluid recruitment, driven by a negative thermal balance.^27^ Despite its superior intradialytic hemodynamic profile, C-HD ranked second in the frequency of real-time reported symptoms (*n* = 6/24), half of which were BP-related. As reported earlier, “feeling cold” was reported more frequently and with greater severity during C-HD, whereas all other symptoms were unaffected by dialysis modality.^15^ Overall, the relationship between IDH-susceptibility and symptoms appeared consistent across all modalities.

The most important strength of this study is the prospective collection of both BP readings and adverse symptoms, according to a stringent predefined study protocol. This approach is unique because it compares the most widely used IDH criteria (from the KDOQI and EBPG guidelines) with the threshold-based definition, which is most strongly linked to survival. In addition, BP was measured twice as frequent as in routine daily practice (every 15 minutes) and symptoms were not only assessed real time, but also by completing queries on patient-reported outcome measures. Other less common but even highly weighty strengths include the training instructions shortly before inclusion and the 100% completion rate of the questionnaires as well as the participants’ willingness to fill in the surveys immediately after the last study session. All these factors may have helped to minimize recall bias for the most part in this patient group.^28^^,^^29^

Nevertheless, several limitations should be acknowledged. First, the limited power due to both the small sample size and low incidence of symptoms and IDH may have constrained our ability to detect more subtle correlations between IDH and symptoms. Second, the predominance of Caucasian patients, the relatively short dialysis vintage, and young age of our cohort may restrict the generalizability of our findings and potentially underestimate the true burden of symptomatic IDH in older or more high-risk populations. In addition, the requirement to achieve a convection volume ≥ 23 L per treatment might have introduced selection bias, although both prospective and retrospective studies have recently shown that high-volume HDF can persistently be achieved in the vast majority of dialysis patients.^30^^,^^31^ Third, the analysis of patient groups based on IDH-susceptibility was not prespecified and should be considered exploratory. Nevertheless, the multiple BP readings within each patient enabled differentiation between IDH-susceptible and nonsusceptible individuals. Lastly, despite content validity and test-retest reliability of the original DSI, the mDSI may require additional validation.

In conclusion, the most striking outcome of this study is the finding that by far, most IDH episodes occur asymptomatic. In fact, these findings indicate that aIDH remains undetected as long as symptom-based definitions are used. Given our observation that the IDH-frequency increases toward the end of sessions, extra BP readings in the latter part of the treatment could be beneficial, particularly for IDH-prone patients. Second, only half of the real-time occurring adverse symptoms appeared related to IDH. These findings are consistent with our third observation, namely that PID-PROMs do not differ between groups with varying IDH-susceptibility.

Clinically, our study may have important consequences. Because adverse symptoms are frequently attributed to IDH, in daily practice, pump-speed is reduced and/or fluid is administered. As a result, unnecessary undertreatment and/or fluid overload may occur. Given that frequent cuff-BP monitoring can be burdensome for patients, future research should explore new technologies, such as artificial intelligence–driven risk stratification and real-time prediction tools, and continuous BP monitoring techniques. Early detection of potentially harmful IDH episodes may enable personalized treatment policies, ultimately improving the outcome of our patients.

## Disclosure

M.J.N., M.P.C.G., and P.A.R. report unrestricted grant support, paid to the institution, from Niercentrum aan de Amstel, Elyse Klinieken, the Netherlands, and B. Braun Avitum AG, Melsungen, Germany. All other authors declared no competing interests. B. Braun Avitum AG and Niercentrum aan de Amstel had no involvement in the collection, analysis and interpretation of data, or in the reporting of the results. Members of the study team are not employed by B. Braun Avitum AG or Niercentrum aan de Amstel.

## Supplementary Material

Supplementary File (PDF) **Figure S1.** Study scheme. **Figure S2.** Blood pressure and real-time symptom assessment. **Figure S3.** Study flowchart. **Figure S4.** Overview of the IDH frequency and the corresponding symptom burden. **Table S1.** Modified Dialysis Symptom Index. **Table S2.** Dialysis treatment characteristics. **Table S3.** Frequency of symptomatic and asymptomatic IDH stratified by dialysis modality. **Table S4.** IDH and adverse symptoms stratified by dialysis modality. **Table S5.** Patient characteristics after stratification into tertiles of IDH susceptibility. **Table S6.** IDH susceptibility and PID-PROMs stratified by dialysis modality. **Table S7.** IDH susceptibility and symptom severity (PID-PROMs) stratified by dialysis modality. **Table S8.** IDH susceptibility and PID-PROMs, as indicated by the total number of symptoms as well as symptom burden, stratified by dialysis modality. **Table S9.** STROBE Checklist.

## Footnotes

Supplementary File (PDF)
**Figure S1.** Study scheme. **Figure S2.** Blood pressure and real-time symptom assessment. **Figure S3.** Study flowchart. **Figure S4.** Overview of the IDH frequency and the corresponding symptom burden. **Table S1.** Modified Dialysis Symptom Index. **Table S2.** Dialysis treatment characteristics. **Table S3.** Frequency of symptomatic and asymptomatic IDH stratified by dialysis modality. **Table S4.** IDH and adverse symptoms stratified by dialysis modality. **Table S5.** Patient characteristics after stratification into tertiles of IDH susceptibility. **Table S6.** IDH susceptibility and PID-PROMs stratified by dialysis modality. **Table S7.** IDH susceptibility and symptom severity (PID-PROMs) stratified by dialysis modality. **Table S8.** IDH susceptibility and PID-PROMs, as indicated by the total number of symptoms as well as symptom burden, stratified by dialysis modality. **Table S9.** STROBE Checklist.
