# Supplementary material for: Intradialytic Hypotensive Episodes are Only Occasionally Associated With Adverse Symptoms
Source: Kidney Int Rep. 2025 Dec 12;11(3):103718. doi: 10.1016/j.ekir.2025.103718 (PMC12890802; doi:10.1016/j.ekir.2025.103718)
Supplement: Supplementary File (PDF) — Figure S1. Study scheme. Figure S2. Blood pressure and real-time symptom assessment. Figure S3. Study flowchart. Figure S4. Overview of the IDH frequency and the corresponding symptom burden. Table S1. Modified Dialysis Symptom Index. Table S2. Dialysis treatment characteristics. Table S3. Frequency of symptomatic and asymptomatic IDH stratified by dialysis modality. Table S4. IDH and adverse symptoms stratified by dialysis modality. Table S5. Patient characteristics after stratification into tertiles of IDH susceptibility. Table S6. IDH susceptibility and PID-PROMs stratified by dialysis modality. Table S7. IDH susceptibility and symptom severity (PID-PROMs) stratified by dialysis modality. Table S8. IDH susceptibility and PID-PROMs, as indicated by the total number of symptoms as well as symptom burden, stratified by dialysis modality. Table S9. STROBE Checklist. [file mmc1.pdf]

Supplementary material

Figure S1. Study scheme

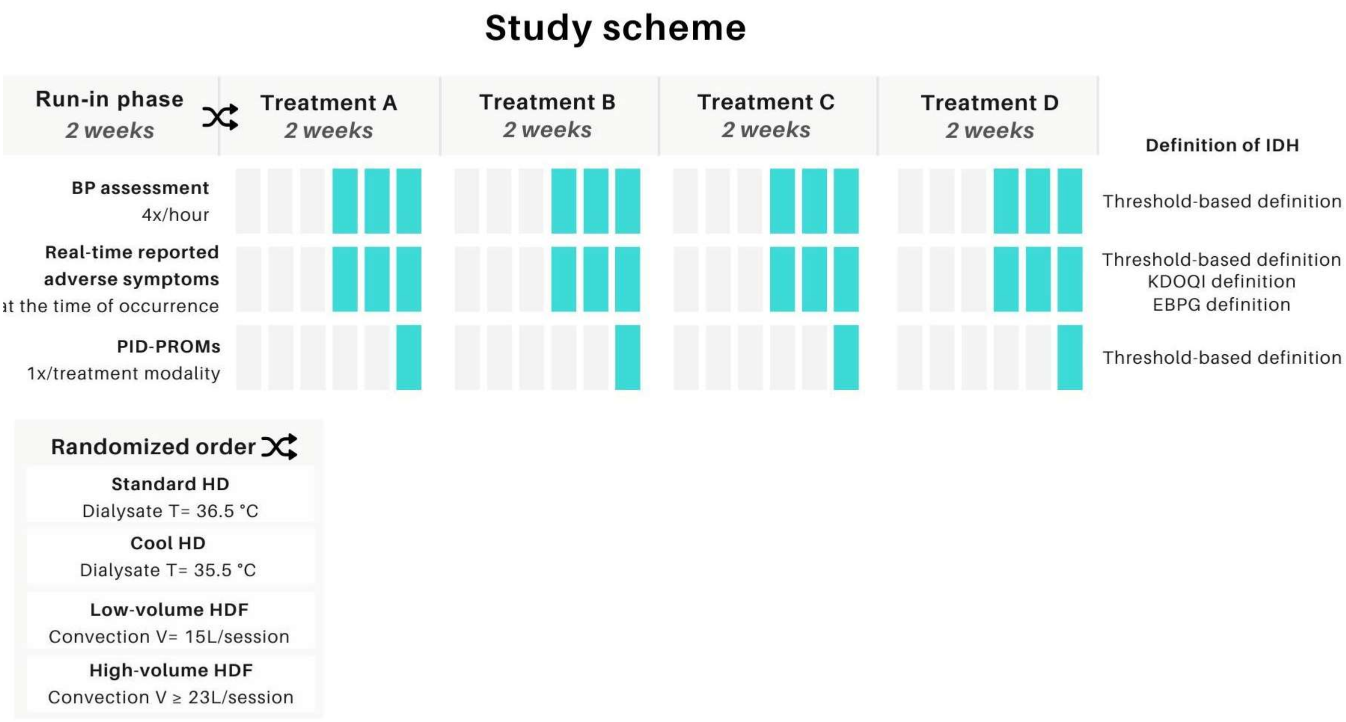

Overview of study scheme. The run-in phase (total duration of 2 weeks) is followed by four different treatment modalities (S-HD, C-HD, LV-HDF, HV-HDF) in a randomized order. The blue blocks represent the treatment sessions during which BP, real-time symptoms or PID-PROMs were assessed. The IDH definitions used for each assessment are shown on the right.

Abbreviations: IDH, intradialytic hypotension; BP, blood pressure; PID-PROMs, physical intradialytic outcome measures; HD, hemodialysis; T, temperature; HDF, hemodiafiltration; V, volume; threshold-based definition: SBP<90 mmHg or <100 mmHg with a pre-dialysis SBP<160 mmHg or SBP≥160 mmHg, respectively; KDOQI definition, National Kidney Disease Outcomes Quality Initiative (KDOQI) guideline definition: SBP-decrease ≥20mmHg or MAP-decrease ≥10 mmHg accompanied by symptoms; EBPG definition, European Best Practice Guideline definition: KDOQI description + intervention.

Figure S2. Blood pressure and real-time symptom assessment

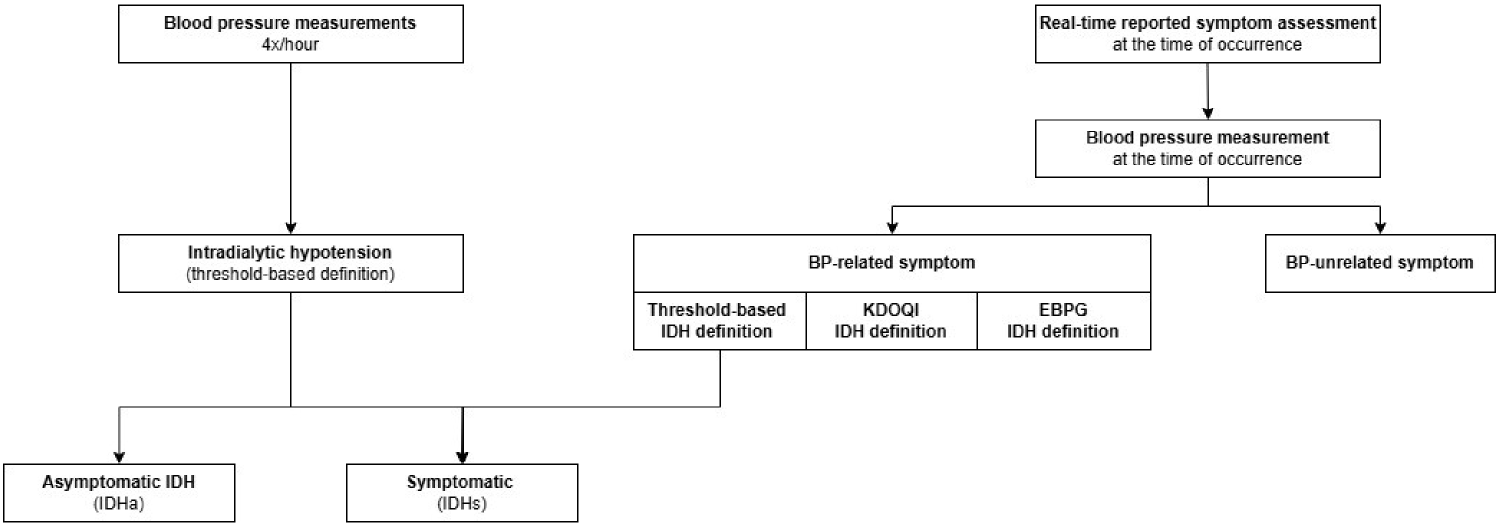

Overview of blood pressure (BP) and real-time reported symptom assessment. Symptomatic and asymptomatic IDH occurrence were assessed using the threshold-based IDH-definition. Real-time reported symptoms were classified as BP-related or BP-unrelated based on the concurrent presence of IDH, as defined by the threshold-based, KDOQI and/or EBPG criteria. *Abbreviations*: IDH, intradialytic hypotension; BP, blood pressure; PID-PROMs, physical intradialytic outcome measures; threshold-based definition: SBP<90 mmHg or <100 mmHg with a pre-dialysis SBP<160 mmHg or SBP≥160 mmHg, respectively; KDOQI definition, National Kidney Disease Outcomes Quality Initiative (KDOQI) guideline definition: SBP-decrease ≥20mmHg or MAP-decrease ≥10 mmHg accompanied by symptoms; EBPG definition, European Best Practice Guideline definition: KDOQI description + intervention.

Table S1. Modified Dialysis Symptom Index (mDSI)

During the past week: did you experience this symptom during dialysis?	Not at all	A little bit	Some what	Quite a bit	Very much
1. Dizziness or light-headedness.	0	1	2	3	4
2. Nausea	0	1	2	3	4
3. Vomiting	0	1	2	3	4
4. Headache	0	1	2	3	4
5. Muscle cramps	0	1	2	3	4
6. Swelling of the legs.	0	1	2	3	4
7. Shortness of breath	0	1	2	3	4
8. Chest pain	0	1	2	3	4
9. Itching	0	1	2	3	4
10. Feeling cold	0	1	2	3	4
11. Shivering	0	1	2	3	4
12. Feeling tired or lack of energy	0	1	2	3	4
13. Recovery time after dialysis:<br>0= none<br>1= after 1 hour<br>2= after half a day<br>3= the next day<br>4= the day of the next dialysis	0	1	2	3	4

Figure S3. Study flowchart

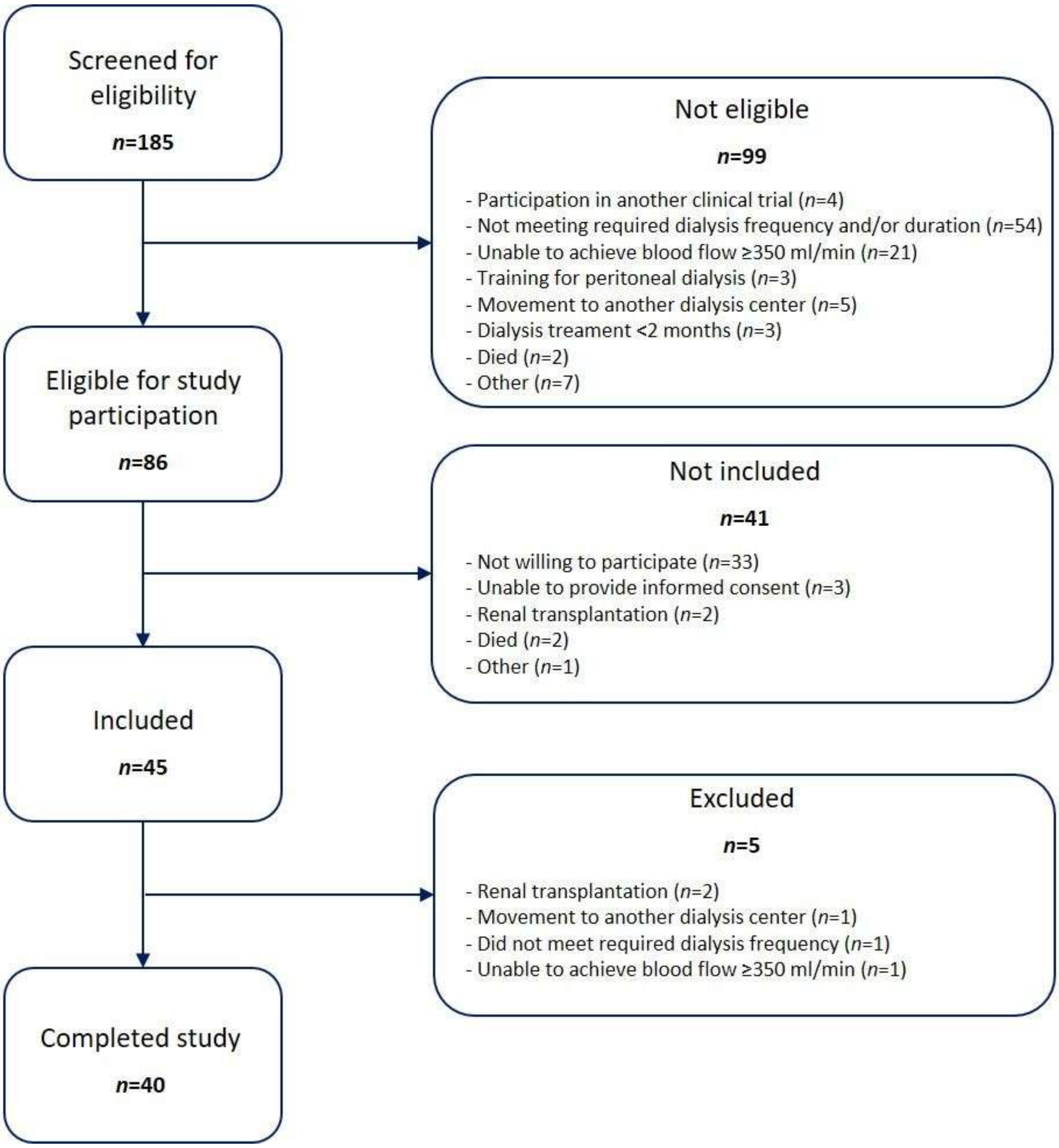

Table S2. Dialysis treatment characteristics

Treatment parameter	Dialysis modality			
	**S-HD**	**C-HD**	**LV-HFDF**	**HV-HDF**
Delivered treatment time (min)	237	235	236	235
Blood flow (mL/min)	341 ± 63	329 ± 53	336 ± 48	345 ± 43
Ultrafiltration volume (L/treatment)	2.3 ± 0.8	2.3 ± 0.8	2.4 ± 0.9	2.3 ± 0.8
Convection volume (L/ treatment)	n/a	n/a	15.5 ± 3.9	22.0 ± 2.8

Values are presented as number (%) or mean ± standard deviation.
Abbreviations: S-HD, standard hemodialysis; C-HD, cooled hemodialysis; LV-HDF, low-volume hemodiafiltration; HV-HDF, high-volume hemodiafiltration; n/a, not applicable.

Table S3. Frequency of symptomatic and asymptomatic IDH stratified by dialysis modality

Intradialytic hypotension (threshold-based definition)			
**Dialysis modality**	**Asymptomatic IDH**	**Symptomatic IDH**	**Total**
S-HD	93	3	96
C-HD	31	0	31
LV-HDF	59	0	59
HV-HDF	35	1	36
Total	*218*	*4*	*222*

Distribution of symptomatic and asymptomatic IDH across dialysis modalities. IDH was defined as SBP<90 mmHg or <100 mmHg with a pre-dialysis systolic blood pressure <160 mmHg or ≥160 mmHg, respectively.
Abbreviations: IDH, intradialytic hypotension; S-HD, standard hemodialysis; C-HD, cooled hemodialysis; LV-HDF, low-volume hemodiafiltration; HV-HDF, high-volume hemodiafiltration.

Table S4. IDH and adverse symptoms stratified by dialysis modality

Real-time intradialytic symptoms					
**Dialysis modality**	**Hypotension with symptoms**			**Symptoms without hypotension**	**Total**
	**Threshold-based**	**KDOQI**	**EBPG**		
S-HD	3 (30%)	5 (50%)	5 (50%)	5 (50%)	10
C-HD	0 (0%)	3 (50%)	3 (50%)	3 (50%)	6
LV-HDF	0 (0%)	3 (75%)	3 (75%)	1 (25%)	4
HV-HDF	1 (25%)	2 (50%)	2 (50%)	2 (50%)	4
Total	*4*	*13*	*13*	*11*	*24*

Distribution of real-time intradialytic symptoms and their relationship with IDH across dialysis modalities, according to three definitions: threshold-based, KDOQI and EBPG definition.
Abbreviations: IDH, intradialytic hypotension; threshold-based definition: SBP<90 mmHg or <100 mmHg with a pre-dialysis SBP<160 mmHg or SBP≥160 mmHg, respectively; KDOQI definition, National Kidney Disease Outcomes Quality Initiative (KDOQI) guideline definition: SBP-decrease ≥20mmHg or MAP-decrease ≥10 mmHg accompanied by symptoms; EBPG definition, European Best Practice Guideline definition: KDOQI description + intervention; S-HD, standard hemodialysis; C-HD, cooled hemodialysis; LV-HDF, low-volume hemodiafiltration; HV-HDF, high-volume hemodiafiltration.

Table S5. Patient characteristics after stratification into tertiles of IDH-susceptibility

	IDH-resistant (n=13)	Intermediate IDH-prone (n=14)	IDH-prone (n=13)	p-value
**Demographics**				
Male (%)	11 (85)	8 (57)	11 (85)	0.16
Age (years)	68.4 ± 15.0	68.6 ± 9.5	72.2 ± 15.9	0.74
BMI (kg/m^2)	26.0 ± 4.2	26.0 ± 5.1	25.6 ± 4.9	0.96
Smoking status (%)				
Non-smoker	6 (46)	4 (29)	4 (31)	0.59
Former smoker	3 (23)	7 (50)	8 (62)	0.13
Current smoker	4 (31)	3 (21)	1 (8)	0.33
**Medical history**				
Dialysis vintage (years)	5.0 [3.0 – 7.0]	3.0 [2.0 – 5.0]	6.0 [2.0 – 8.0]	0.10
**Comorbid conditions**				
DM (%)	3 (23)	8 (57)	8 (62)	0.10
Hypertension (%)	11 (85)	7 (50)	10 (77)	0.12
History of CVD (%)	7 (54)	12 (86)	10 (77)	0.16
Heart failure	5 (38)	7 (50)	6 (46)	0.83
Myocardial infarction	2 (15)	5 (36)	4 (31)	0.47
Angina pectoris	1 (8)	5 (36)	5 (38)	0.15
CVA	2 (15)	2 (14)	1 (8)	0.81
TIA	0 (0)	1 (7)	3 (23)	0.13
PAOD	1 (8)	5 (36)	3 (23)	0.22

Values are presented as number (*n*) (%) for categorical variables, and mean (± SD) or median (IQR 25%-75%) for continuous variables.

Abbreviations: IDH, intradialytic hypotension; BMI, body mass index; DM, diabetes mellitus; CVD, cardiovascular disease; CVA, cerebral vascular accident; TIA, transient ischemic attack; PAOD, peripheral arterial occlusive disease.

Figure S4. Overview of the IDH frequency and the corresponding symptom burden

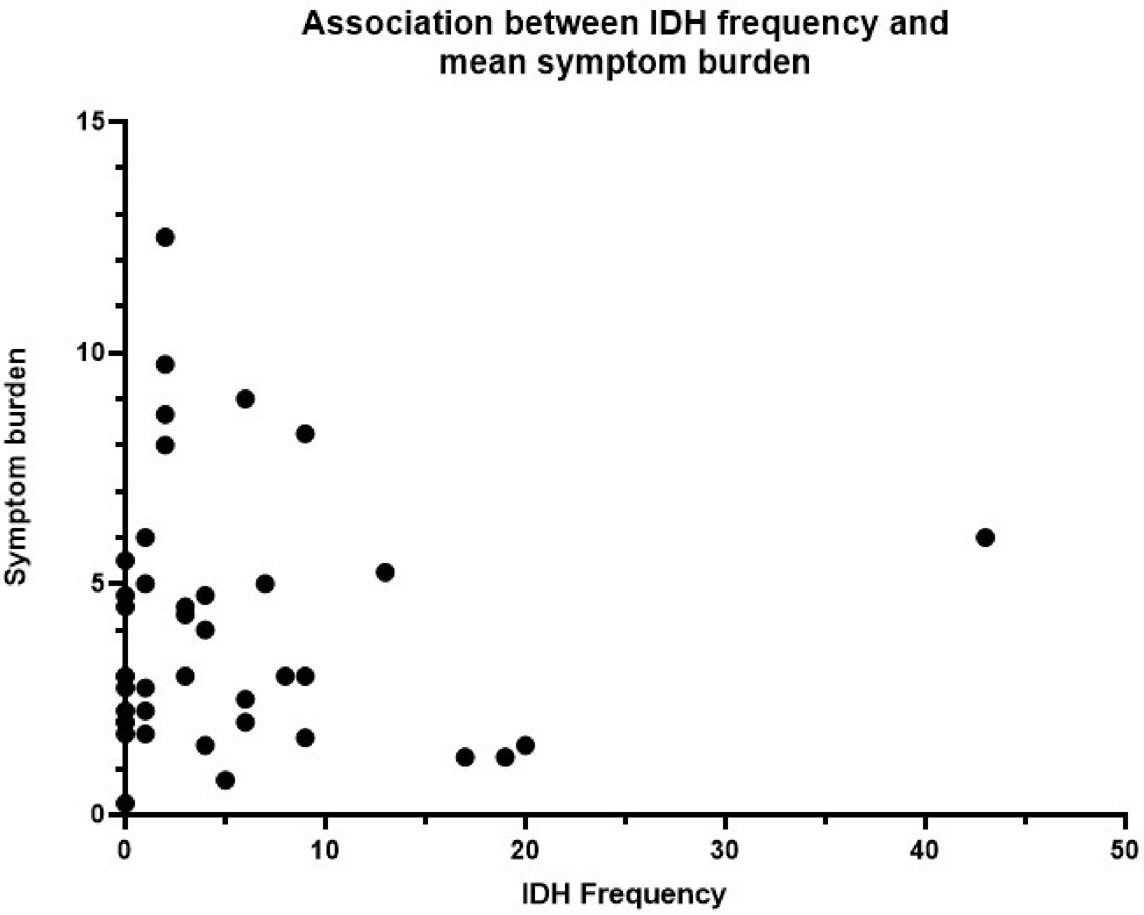

Each data point represents the IDH frequency during the study (i.e. twelve sessions) and the corresponding averaged symptom burden score (ranging from 0-48).
Abbreviations: IDH, intradialytic hypotension.

Table S6. IDH-susceptibility and PID-PROMs stratified by dialysis modality

Frequency of PID-PROMs				
**PID-PROMs**	**IDH-resistant** (n=13)	**Intermediate IDH-prone** (n=14)	**IDH-prone** (n=13)	**Total**
**Dizziness**				
S-HD	3/12 (25%)	3/14 (21%)	4/12 (33%)	10
C-HD	2/12 (17%)	3/13 (23%)	3/13 (23%)	8
LV-HDF	0/13 (0%)	6/14 (43%)	4/10 (40%)	10
HV-HDF	2/13 (15%)	3/13 (23%)	3/11 (27%)	8
**Nausea**				
S-HD	1/12 (8%)	1/14 (7%)	1/12 (8%)	3
C-HD	0/12 (0%)	1/13 (8%)	0/13 (0%)	1
LV-HDF	0/13 (0%)	2/14 (14%)	2/10 (20%)	4
HV-HDF	1/13 (8%)	1/13 (8%)	0/11 (15%)	2
**Vomiting**				
S-HD	0/12 (0%)	2/14 (14%)	0/12 (0%)	2
C-HD	0/12 (0%)	0/13 (0%)	0/13 (0%)	0
LV-HDF	0/13 (0%)	0/14 (0%)	0/10 (0%)	0
HV-HDF	0/13 (0%)	0/13 (0%)	0/11 (0%)	0
**Headache**				
S-HD	1/12 (8%)	4/14 (29%)	7/12 (58%)	12
C-HD	2/12 (17%)	2/13 (14%)	4/13 (31%)	8
LV-HDF	1/13 (8%)	3/14 (21%)	5/10 (50%)	9
HV-HDF	3/13 (23%)	2/13 (14%)	3/11 (27%)	8
**Muscle cramps**				
S-HD	1/12 (8%)	9/14 (64%)	7/12 (58%)	17
C-HD	2/12 (17%)	5/13 (38%)	6/13 (46%)	13
LV-HDF	5/13 (39%)	9/14 (64%)	3/10 (30%)	17
HV-HDF	5/13 (39%)	7/13 (54%)	3/11 (27%)	15
**Swelling of the legs**				
S-HD	1/12 (8%)	4/14 (29%)	1/12 (8%)	6
C-HD	0/12 (0%)	2/13 (14%)	2/13 (14%)	4
LV-HDF	0/13 (0%)	2/14 (14%)	1/10 (10%)	3
HV-HDF	0/13 (0%)	1/13 (8%)	3/11 (27%)	4
**Shortness of breath**				
S-HD	1/12 (8%)	3/14 (21%)	1/12 (8%)	5
C-HD	0/12 (0%)	1/13 (8%)	3/13 (23%)	4
LV-HDF	2/13 (15%)	3/14 (21%)	3/10 (30%)	8
HV-HDF	2/13 (15%)	1/13 (8%)	0/11 (0%)	3
**Chest pain**				
S-HD	1/12 (8%)	1/14 (7%)	1/12 (8%)	3
C-HD	2/12 (17%)	0/13 (0%)	0/13 (0%)	2
LV-HDF	2/13 (15%)	0/14 (0%)	2/10 (20%)	4
HV-HDF	1/13 (8%)	1/13 (8%)	0/11 (0%)	2

**Itching**				
S-HD	4/12 (33%)	7/14 (50%)	4/12 (33%)	15
C-HD	5/12 (42%)	3/13 (23%)	3/13 (23%)	11
LV-HDF	5/13 (39%)	9/14 (64%)	3/10 (30%)	17
HV-HDF	4/13 (31%)	4/13 (31%)	2/11 (18%)	10
**Feeling cold**				
S-HD	4/12 (33%)	9/14 (64%)	6/12 (50%)	19
C-HD	8/12 (67%)	11/13 (85%)	6/13 (46%)	25
LV-HDF	5/13 (38%)	8/14 (57%)	6/10 (60%)	19
HV-HDF	6/13 (46%)	7/13 (54%)	3/11 (27%)	16
**Shivering**				
S-HD	2/12 (17%)	4/14 (29%)	1/12 (8%)	7
C-HD	6/12 (50%)	3/13 (23%)	2/13 (15%)	11
LV-HDF	2/13 (15%)	3/14 (21%)	3/10 (30%)	8
HV-HDF	3/13 (23%)	3/13 (23%)	1/11 (9%)	7
**Feeling tired or lack of energy**				
S-HD	5/12 (42%)	7/14 (50%)	6/12 (50%)	18
C-HD	7/12 (58%)	8/13 (62%)	7/14 (54%)	22
LV-HDF	6/13 (46%)	6/14 (43%)	7/10 (70%)	19
HV-HDF	8/13 (62%)	7/13 (54%)	7/11 (64%)	22

Data are presented as number of patients (%) experiencing the symptoms.
Groups are sorted by IDH incidence.
Abbreviations: PID-PROMs, physical intradialytic patient-reported outcome measures; IDH, intradialytic hypotension; S-HD, standard hemodialysis; C-HD, cooled hemodialysis; LV-HDF, low-volume hemodiafiltration; HV-HDF, high-volume hemodiafiltration.

Table S7. IDH-susceptibility and symptom severity (PID-PROMs) stratified by dialysis modality

Severity of PID-PROMs				
**PID-PROMs**	**IDH-resistant** (n=13)	**Intermediate IDH-prone** (n=14)	**IDH-prone** (n=13)	**Total**
**Dizziness**				
S-HD	1.0 (1.0-1.0)	1.0 (1.0-1.0)	2.0 (1.0-4.0)	1.0 (1.0-4.0)
C-HD	1.0 (1.0-1.0)	1.0 (1.0-2.0)	2.0 (1.0-2.0)	1.0 (1.0-2.0)
LV-HDF	1.0 (1.0-1.0)	1.0 (1.0-2.0)	2.0 (1.0-2.0)	1.0 (1.0-2.0)
HV-HDF	1.0 (1.0-1.0)	1.0 (1.0-2.0)	2.0 (1.0-2.0)	1.0 (1.0-2.0)
**Nausea**				
S-HD	2.0 (2.0-2.0)	1.0 (1.0-1.0)	1.0 (1.0-1.0)	1.0 (1.0-2.0)
C-HD	n/a	1.0 (1.0-1.0)	n/a	1.0 (1.0-1.0)
LV-HDF	n/a	1.0 (1.0-1.0)	1.5 (1.0-2.0)	1.0 (1.0-2.0)
HV-HDF	2.0 (2.0-2.0)	1.0 (1.0-1.0)	n/a	1.0 (1.0-2.0)
**Vomiting**				
S-HD	n/a	1.5 (1.0-2.0)	n/a	1.5 (1.0-2.0)
C-HD	n/a	n/a	n/a	n/a
LV-HDF	n/a	n/a	n/a	n/a
HV-HDF	n/a	n/a	n/a	n/a
**Headache**				
S-HD	1.0 (1.0-1.0)	1.0 (1.0-1.0)	1.0 (1.0-1.0)	1.0 (1.0-3.0)
C-HD	1.0 (1.0-1.0)	2.5 (1.0-4.0)	1.0 (1.0-2.0)	1.0 (1.0-4.0)
LV-HDF	1.0 (1.0-1.0)	1.0 (1.0-2.0)	1.0 (1.0-1.0)	1.0 (1.0-2.0)
HV-HDF	1.0 (1.0-3.0)	1.5 (1.0-2.0)	1.0 (1.0-1.0)	1.0 (1.0-3.0)
**Muscle cramps**				
S-HD	1.0 (1.0-1.0)	1.0 (1.0-3.0)	1.0 (1.0-4.0)	1.0 (1.0-4.0)
C-HD	1.0 (1.0-1.0)	1.0 (1.0-3.0)	1.0 (1.0-1.0)	1.0 (1.0-3.0)
LV-HDF	1.0 (1.0-1.0)	1.0 (1.0-4.0)	1.0 (1.0-1.0)	1.0 (1.0-4.0)
HV-HDF	1.0 (1.0-2.0)	1.0 (1.0-2.0)	1.0 (1.0-1.0)	1.0 (1.0-2.0)
**Swelling of the legs**				
S-HD	1.0 (1.0-1.0)	1.0 (1.0-2.0)	1.0 (1.0-1.0)	1.0 (1.0-2.0)
C-HD	n/a	2.0 (2.0-2.0)	1.0 (1.0-1.0)	1.5 (1.0-2.0)
LV-HDF	n/a	1.0 (1.0-1.0)	1.0 (1.0-1.0)	1.0 (1.0-1.0)
HV-HDF	n/a	1.0 (1.0-1.0)	1.0 (1.0-1.0)	1.0 (1.0-1.0)
**Shortness of breath**				
S-HD	1.0 (1.0-1.0)	1.0 (1.0-1.0)	1.0 (1.0-1.0)	1.0 (1.0-1.0)
C-HD	n/a	2.0 (2.0-2.0)	1.0 (1.0-1.0)	1.0 (1.0-2.0)
LV-HDF	1.5 (1.0-2.0)	1.0 (1.0-2.0)	1.0 (1.0-3.0)	1.0 (1.0-3.0)
HV-HDF	1.0 (1.0-1.0)	2.0 (2.0-2.0)	n/a	1.0 (1.0-2.0)
**Chest pain**				
S-HD	1.0 (1.0-1.0)	1.0 (1.0-1.0)	1.0 (1.0-1.0)	1.0 (1.0-1.0)
C-HD	1.0 (1.0-1.0)	n/a	n/a	1.0 (1.0-1.0)
LV-HDF	1.0 (1.0-3.0)	n/a	1.0 (1.0-1.0)	1.0 (1.0-3.0)
HV-HDF	1.0 (1.0-1.0)	1.0 (1.0-1.0)	n/a	1.0 (1.0-1.0)

**Itching**				
S-HD	1.0 (1.0-2.0)	1.0 (1.0-2.0)	1.0 (1.0-4.0)	1.0 (1.0-4.0)
C-HD	1.0 (1.0-2.0)	1.0 (1.0-2.0)	1.0 (1.0-1.0)	1.0 (1.0-2.0)
LV-HDF	1.0 (1.0-2.0)	1.0 (1.0-3.0)	1.0 (1.0-2.0)	1.0 (1.0-3.0)
HV-HDF	1.0 (1.0-3.0)	1.0 (1.0-3.0)	1.0 (1.0-1.0)	1.0 (1.0-3.0)
**Feeling cold**				
S-HD	1.0 (1.0-1.0)	1.0 (1.0-4.0)	1.0 (1.0-2.0)	1.0 (1.0-4.0)
C-HD	1.5 (1.0-3.0)	2.0 (1.0-4.0)	1.5 (1.0-2.0)	2.0 (1.0-4.0)
LV-HDF	1.0 (1.0-3.0)	1.0 (1.0-2.0)	1.0 (1.0-3.0)	1.0 (1.0-3.0)
HV-HDF	1.0 (1.0-2.0)	1.0 (1.0-4.0)	1.0 (1.0-2.0)	1.0 (1.0-4.0)
**Shivering**				
S-HD	1.5 (1.0-2.0)	1.5 (1.0-2.0)	1.0 (1.0-1.0)	1.0 (1.0-2.0)
C-HD	1.0 (1.0-1.0)	1.0 (1.0-1.0)	1.5 (1.0-2.0)	1.0 (1.0-2.0)
LV-HDF	1.0 (1.0-1.0)	2.0 (1.0-2.0)	2.0 (1.0-2.0)	1.5 (1.0-2.0)
HV-HDF	1.0 (1.0-1.0)	2.0 (1.0-2.0)	1.0 (1.0-1.0)	1.0 (1.0-2.0)
**Feeling tired or lack of energy**				
S-HD	2.0 (1.0-3.0)	2.0 (1.0-4.0)	1.0 (1.0-3.0)	2.0 (1.0-4.0)
C-HD	1.0 (1.0-4.0)	2.0 (1.0-4.0)	1.0 (1.0-2.0)	1.5 (1.0-4.0)
LV-HDF	2.0 (1.0-4.0)	2.0 (1.0-4.0)	1.0 (1.0-4.0)	2.0 (1.0-4.0)
HV-HDF	1.0 (1.0-4.0)	1.0 (1.0-3.0)	1.0 (1.0-3.0)	1.0 (1.0-4.0)
**Recovery time**				
S-HD	1.0 (0.0-3.0)	2.0 (0.0-4.0)	1.0 (0.0-2.0)	1.0 (0.0-4.0)
C-HD	1.5 (0.0-3.0)	1.0 (0.0-4.0)	1.0 (0.0-2.0)	1.0 (0.0-4.0)
LV-HDF	1.0 (0.0-3.0)	2.0 (0.0-4.0)	1.0 (0.0-4.0)	1.0 (0.0-4.0)
HV-HDF	1.0 (0.0-3.0)	2.0 (0.0-4.0)	1.0 (0.0-3.0)	1.0 (0.0-4.0)

Data are presented as median severity score (range: minimum-maximum).
Groups are sorted by IDH incidence.
Abbreviations: PID-PROMs, physical intradialytic patient-reported outcome measures; IDH, intradialytic hypotension; S-HD, standard hemodialysis; C-HD, cooled hemodialysis; LV-HDF, low-volume hemodiafiltration; HV-HDF, high-volume hemodiafiltration; n/a, not applicable.

Table S8. IDH-susceptibility and PID-PROMs, as indicated by the total number of symptoms as well as symptom burden, stratified by dialysis modality

PID-PROMs	**IDH-resistant** (n=13)	**Intermediate IDH-prone** (n=14)	**IDH-prone** (n=13)	**Total**
**Total number of symptoms**				
S-HD	2.0 (1.0-3.5)	3.5 (2.0-5.0)	3.0 (1.5-4.5)	3.0 (2.0-5.0)
C-HD	3.0 (2.0-4.5)	3.0 (1.8-5.0)	3.0 (1.0-4.5)	3.0 (2.0-5.0)
LV-HDF	2.0 (1.0-3.0)	4.0 (2.0-5.3)	5.0 (2.0-9.5)	4.0 (2.0-7.0)
HV-HDF	3.0 (1.0-4.5)	2.0 (1.0-4.5)	3.0 (1.0-5.0)	3.0 (1.0-6.0)
**Overall symptom severity score**				
S-HD	1.0 (1.0-1.7)	1.1 (1.0-1.8)	1.0 (1.0-1.3)	1.0 (1.0-1.7)
C-HD	1.4 (1.0-1.6)	2.0 (1.1-2.0)	1.1 (1.0-1.5)	1.4 (1.0-2.0)
LV-HDF	1.0 (1.0-1.6)	1.6 (1.3-2.0)	1.0 (1.0-1.3)	1.1 (1.0-1.8)
HV-HDF	1.3 (1.0-1.5)	1.1 (1.0-1.6)	1.0 (1.0-1.1)	1.0 (1.0-1.4)
**Symptom burden**				
S-HD	2.0 (1.0-5.0)	4.0 (2.0-9.3)	3.5 (1.3-5.0)	3.5 (2.0-5.0)
C-HD	3.5 (2.0-6.8)	5.0 (2.5-6.5)	4.0 (1.0-5.0)	4.0 (2.0-6.0)
LV-HDF	2.0 (1.0-6.0)	6.5 (2.8-7.0)	4.0 (1.8-8.0)	4.0 (2.0-7.0)
HV-HDF	3.0 (1.0-6.5)	2.0 (1.0-5.5)	1.0 (1.0-4.0)	2.0 (1.0-5.0)

Data are presented as median (IQR).
Groups are sorted by IDH incidence.
Abbreviations: PID-PROMs, physical intradialytic patient-reported outcome measures; IDH, intradialytic hypotension; S-HD, standard hemodialysis; C-HD, cooled hemodialysis; LV-HDF, low-volume hemodiafiltration; HV-HDF, high-volume hemodiafiltration; IQR, interquartile range.

Table S9. STROBE checklist

	Item No	Recommendation	Page No
**Title and abstract**	1	(*a*) Indicate the study's design with a commonly used term in the title or the abstract	2
		(*b*) Provide in the abstract an informative and balanced summary of what was done and what was found	2, 3
**Introduction**			
Background/rationale	2	Explain the scientific background and rationale for the investigation being reported	4, 5
Objectives	3	State specific objectives, including any prespecified hypotheses	5
**Methods**			
Study design	4	Present key elements of study design early in the paper	6
Setting	5	Describe the setting, locations, and relevant dates, including periods of recruitment, exposure, follow-up, and data collection	6-10
Participants	6	(*a*) Give the eligibility criteria, and the sources and methods of selection of participants. Describe methods of follow-up	6, 7
		(*b*) For matched studies, give matching criteria and number of exposed and unexposed	n/a
Variables	7	Clearly define all outcomes, exposures, predictors, potential confounders, and effect modifiers. Give diagnostic criteria, if applicable	7-10
Data sources/ measurement	8*	For each variable of interest, give sources of data and details of methods of assessment (measurement). Describe comparability of assessment methods if there is more than one group	7-10
Bias	9	Describe any efforts to address potential sources of bias	
Study size	10	Explain how the study size was arrived at<br>***Remark:*** *The sample size calculation is not provided here as it was based on the primary outcome of the trial. For details, we referred to the Design paper, see 'Study design and participants' on page 5: " The design of this study, including the sample size calculation, has been described extensively elsewhere.[5]".*	n/a
Quantitative variables	11	Explain how quantitative variables were handled in the analyses. If applicable, describe which groupings were chosen and why	9-11
Statistical methods	12	(*a*) Describe all statistical methods, including those used to control for confounding	10, 11
		(*b*) Describe any methods used to examine subgroups and interactions	10, 11
		(*c*) Explain how missing data were addressed	12
		(*d*) If applicable, explain how loss to follow-up was addressed	n/a
		(*e*) Describe any sensitivity analyses	n/a
**Results**			
Participants	13*	(a) Report numbers of individuals at each stage of study—eg numbers potentially eligible, examined for eligibility,	12, figure S2

		confirmed eligible, included in the study, completing follow-up, and analysed<br>(b) Give reasons for non-participation at each stage<br>(c) Consider use of a flow diagram	 <br>12<br>figure S2
Descriptive data	14*	(a) Give characteristics of study participants (eg demographic, clinical, social) and information on exposures and potential confounders<br>(b) Indicate number of participants with missing data for each variable of interest<br>(c) Summarise follow-up time (eg, average and total amount)	12<br><br>12<br><br>n/a
Outcome data	15*	Report numbers of outcome events or summary measures over time	12-14
Main results	16	(*a*) Give unadjusted estimates and, if applicable, confounder-adjusted estimates and their precision (eg, 95% confidence interval). Make clear which confounders were adjusted for and why they were included<br>(*b*) Report category boundaries when continuous variables were categorized<br>(*c*) If relevant, consider translating estimates of relative risk into absolute risk for a meaningful time period	12-14<br><br>12-14<br><br>n/a
Other analyses	17	Report other analyses done—eg analyses of subgroups and interactions, and sensitivity analyses	13, 14
**Discussion**			
Key results	18	Summarise key results with reference to study objectives	15
Limitations	19	Discuss limitations of the study, taking into account sources of potential bias or imprecision. Discuss both direction and magnitude of any potential bias	17
Interpretation	20	Give a cautious overall interpretation of results considering objectives, limitations, multiplicity of analyses, results from similar studies, and other relevant evidence	15-18
Generalisability	21	Discuss the generalisability (external validity) of the study results	17
**Other information**			
Funding	22	Give the source of funding and the role of the funders for the present study and, if applicable, for the original study on which the present article is based	19
